# VEP examination with new portable device

**DOI:** 10.1007/s10633-022-09911-w

**Published:** 2022-11-27

**Authors:** Miroslav Kuba, Jan Kremláček, František Vít, Zuzana Kubová, Jana Langrová, Jana Szanyi, Marie Chutná

**Affiliations:** Electrophysiological Lab, Dept. of Pathophysiology, Faculty of Medicine, Charles Univ, Simkova 870, 500 03 Hradec Králové, Czech Republic

**Keywords:** VEP portable device, Pattern-reversal, Motion-onset, Cognitive ERP, VEPpeak, VEP diagnostics

## Abstract

**Introduction:**

We developed a new portable device called “VEPpeak” for the examination of visual evoked potentials (VEPs) to extend VEP examination beyond specialized electrophysiological laboratories and to simplify the use of this objective, noninvasive, and low-cost method for diagnostics of visual and central nervous system dysfunctions.

**Methods:**

VEPpeak consists of a plastic headset with a total weight of 390 g containing four EEG amplifiers, an A/D converter, a control unit, and a visual LED stimulator built in the front, vertically adjustable peak. The device is powered and controlled via USB connection from a standard PC/notebook using custom software for visual stimuli generation and for VEP recording and processing. Up to four electrodes can be placed at any scalp location or in combination with two dry electrodes incorporated into the headset. External visual stimulators, such as a tablet, can be used with synchronization. Feasibility and validation studies were conducted with 86 healthy subjects and 76 neuro-ophthalmological patients including 67 who were during the same session also tested with a conventional VEP system.

**Results:**

VEPpeak recordings to standard (pattern-reversal) and non-standard (motion-onset, red-green alternation) were robust and repeatable and obtained also in immobilized patients. Good comparability of results was achieved between VEPpeak and standard examination. Some systematic differences in peak latencies and amplitudes are consistent with differences in stimulus characteristics of the two compared systems.

**Discussion:**

VEPpeak provides an inexpensive system for clinical use requiring portability. In addition to ISCEV standard VEP protocols, free choice of stimuli and bio-signal recordings make the device universal for many electrophysiological purposes.

**Supplementary Information:**

The online version contains supplementary material available at 10.1007/s10633-022-09911-w.

## Introduction

Visual evoked potential (VEP) examination is almost exclusively dependent on robust equipment of electrophysiological laboratories, which typically cannot be simply transported to places where examination of VEPs is needed. Thus, patients must be transported to specialized laboratories irrespective of their mobility. A simplified flash VEP examination which is sometimes used (e.g., https://lkc.com/products/reteval-2/) does not provide sufficient information about dysfunctions of the visual pathway [1, 2]. Since complete VEP examination may not always be available, VEP diagnostic applications are still rather limited. Nevertheless, VEP examination represents a useful diagnostic tool in ophthalmology and neurology and for the evaluation of CNS functional changes in many other medical specialties. This motivated us to develop a fully portable, inexpensive device that could be used in almost any place in all body positions.

We also sought to enable an effective peripheral visual stimulation [3–5] that would not interfere with the central area of the visual field. This would enable prolonged monitoring of VEPs by examined subjects even during some working activities to recognize possible changes in visual perception and attention typically due to fatigue or medicaments. The commonly used pattern-reversal stimulation usually covers the central part of the visual field (“working area”) since standard pattern stimuli provide smaller VEP amplitudes outside the central ~ 20° of the visual field [6]. This can limit the sensitivity of the VEP examination when the pathology influences only the peripheral visual perception. In some cases, this may concern the magnocellular system or the dorsal stream of the visual pathway [7, 8].

## Methods

**Fig. 1:**
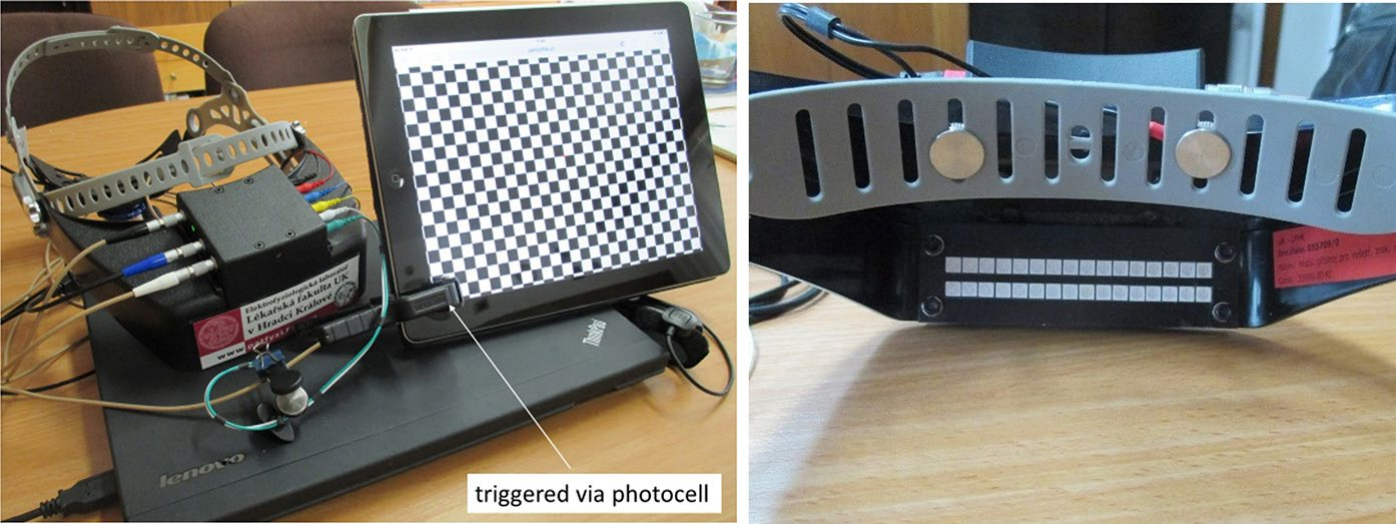
Left—the latest version of the portable VEPpeak device with accessories (control notebook and external stimulator tablet). Right—detailed view on the built-in LED stimulator and two pre-frontal dry recording electrodes (circular targets)

The 5th version of the prototype of a portable device for VEP examination, called “VEPpeak,” developed in our laboratory, was used. It consists of a visual stimulator, 4-channel low-noise EEG amplifiers, and a control unit. It includes a 3D accelerometer for the rejection of head movement artifacts and a surrounding luminance detector for the possibility of adaptive regulation of visual stimuli luminance. Thus, the examination can be performed in various luminance environments. Two digital inputs are available, which can be used to detect the subject’s reactions in cognitive evoked potential examination or external triggering of recordings. All parts are built in a headset (see Fig. 1a), which can be fixed on the head of the examined subject with an adjustable fastener band. The device has a total weight of 390 g and can be connected by a galvanically isolated USB interface with a control and evaluation unit, such as a laptop computer. Special software was developed for visual stimuli generation, recording, and evaluation of VEPs. The device control library is currently available in the Matlab environment. Users can program their own stimuli, start recording with external events and record large spectrum of biosignals.

This version of the device has a CE mark indicating conformity with health, safety, and environmental protection standards, but its aesthetics have not been finalized yet.

### VEP stimuli

#### The built-in visual stimulator

Enables basic VEP examination. It consists of a matrix of 32 color LEDs (diameter of 5 mm) placed in two horizontal rows (2 × 16) in the front part (peak) of the device at about 7.5 cm from the subject’s eyes. Thus, the angular size of each LED is about 4°, and the total stimulus field subtends about 65° × 10°, as shown in Fig. 1b. This arrangement does not allow good visual accommodation (focusing) on small details, mainly in older people (thus for higher pattern spatial frequencies an external stimulator is used—see below), however, it is o.k. with the pattern size of 4° and in motion or color stimuli. Individual LEDs can be controlled with an accuracy of 1 ms by switching them on/off, changing the luminance (in 128 levels), or changing the color. Thus, it is possible to produce flashes, pattern-reversals, pattern on/off, apparent motion, color, or cognitive stimulations. But users can freely program a large spectrum of other visual stimuli. Light fixation points for monocular and binocular stimulations are located in appropriate places. The front part of the device with LEDs is vertically adjustable (can be manually set along the vertical axis), so it is possible to select either stimulation of the central part of the visual field with LEDs in front of the eyes (for neuro-ophthalmological diagnostics) or peripheral stimulation outside the central area of the visual field up to about 40° to the periphery. This might be useful for applications such as the long-term monitoring of VEPs. For this purpose visual motion-stimulation can be used that is effective enough in the periphery [3–5]. A background luminance detector helps for adaptive luminance control of the LEDs to maintain approximately constant luminance contrast related to the luminance of the surroundings.

The following LED stimuli were tested: *Flash stimuli* of 50-ms duration and frequency of 1 Hz (changeable luminance up to about 2.000 cd/m^2^ is available); *“Pattern-reversal”* stimuli (switching the adjacent LEDs on/off) with 1 reversal per second (average luminance of 40 cd/m^2^) provides a spatial frequency of only 0.125 c/deg (LED size dependent); *Motion-onset stimulation* formed by the 200-ms apparent horizontal motion of four LED triplets (luminance of 20 cd/m^2^—the lower luminance corresponds to optimal parameters for the magnocellular pathway activation [3, 8]), which alternate in opposite directions with a temporal frequency of about 5 Hz and a 1-s interstimulus interval (ISI); isoluminant (40 cd/m^2^) *red/green LED stimuli* alternating in the full field with the frequency of 1 Hz. Because of the limited extent of this article visual stimuli for cognitive potentials (P300) examination will be described in a separate article (in preparation).

#### Additional external visual stimulators

**Fig. 2:**
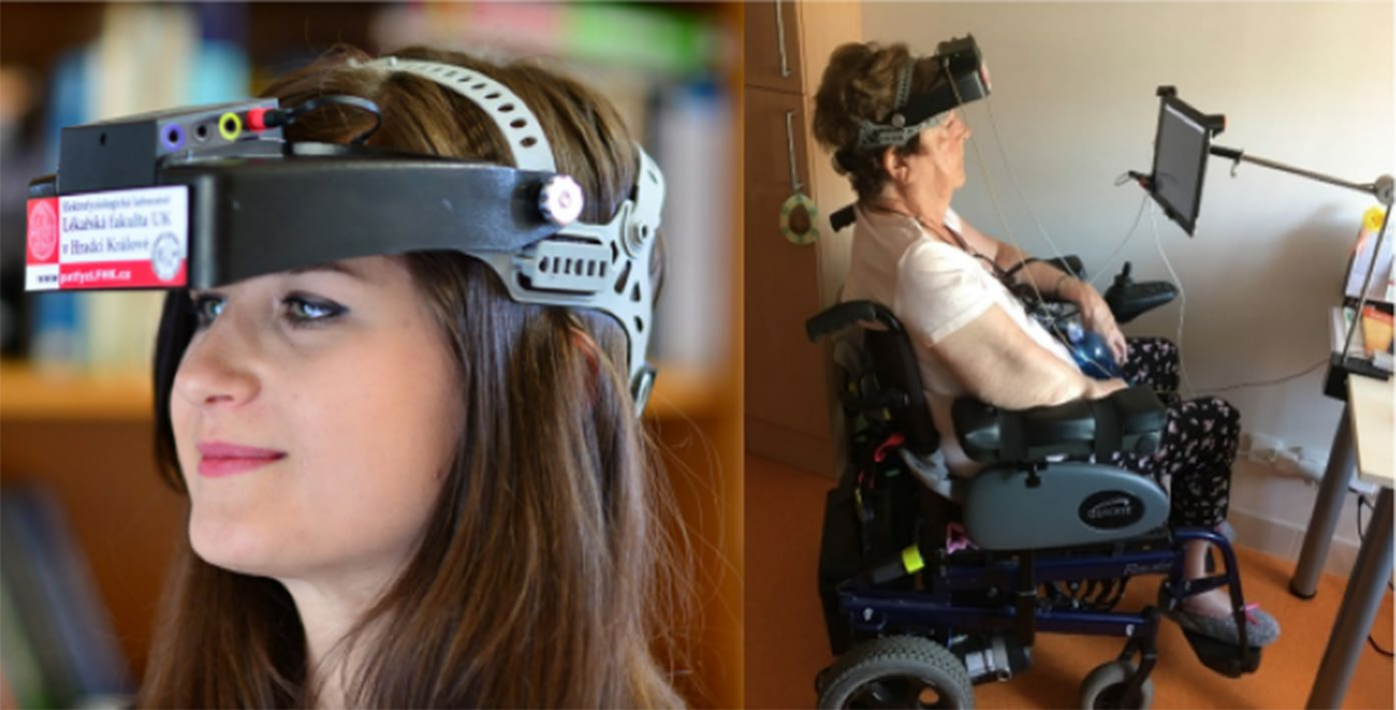
Arrangement of VEP examination with the built-in LED stimulator (left) and in an immobilized patient (right) with the use of the external stimulator in the adjustable holder

During the first clinical tests, it was found that the device with the above-described visual stimulations does not have sufficient diagnostic sensitivity in diagnostics of optic nerve disorders that influence visual acuities, such as Optic Neuritis and Multiple Sclerosis. The reason is that the spatial frequency of the “pattern reversal” produced by the built-in LED stimulator is too low (0.125 c/deg). Replacement of the LEDs with a subtle OLED display or implementation of some optical system that would decrease the size of pattern-reversal elements would be technically difficult and expensive, and we are trying to keep the price of the device low. Thus, the stimulation possibilities were extended by introducing an external stimulator that does not influence the portability of the whole examination set with a weight of 2.5 kg (including the control laptop). A portable low-weight tablet can simply be used—see Figs. 1a and 2. All standard stimuli with comparable parameters according to ISCEV standard [9] (e.g., checkerboard pattern-reversal 60’ and 15’) or any other useful stimuli can be generated in this way. Below described motion-onset stimuli were used according to [8, 10].

The VEP recording is triggered by a photocell that is fixed to the stimulating display and connected to one of the digital inputs of the device. This external stimulator can be placed with the use of an adjustable holder in front of the eyes. The stimulus field of our iPad was about 28° × 21° at a distance of 40 cm from the eyes. It can also be used with immobilized patients (Fig. 2) or patients in bed.

### VEP recording with portable “VEPpeak” device

**Fig. 3:**
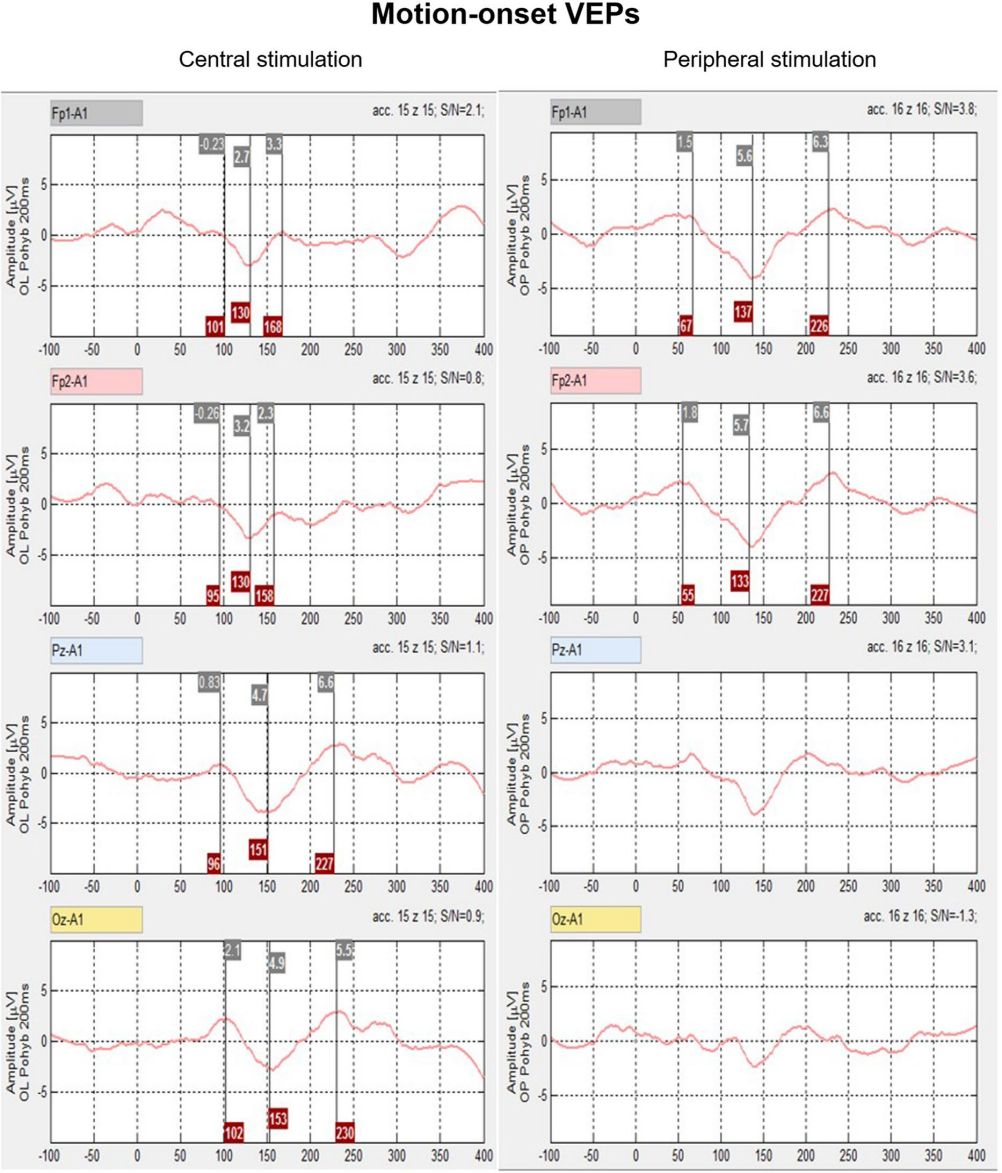
Comparison of the efficiency of central and peripheral motion-onset stimulations in top-two pre-frontal leads and bottom-two parieto-occipital leads Central stimulation activates mainly the occipital and parietal cortex, the stimulation in the lower periphery (outside the central 20°) provides the largest amplitudes at both pre-frontal electrodes

Four unipolar channels can be recorded with dry surface electrodes using built-in EEG low-noise amplifiers (0.8–100 Hz). The attenuation of the high pass filter is 40 dB/decade, and that of the low pass filter is 60 dB/decade). An integrated 16-bit A/D converter with a sampling frequency of 1 kHz is used, and a signal resolution of about 0.1 µV is achieved. Two fixed electrodes are built into the frontal part of the headset fixation band at positions Fp1 and Fp2, which do not require any special montage on a non-hairy forehead. These two channels are ready for immediate, simple, and even non-professional monitoring of VEPs. VEPs are recordable in this location when suitable visual stimuli are used, such as peripheral motion-onset stimulation (see Fig. 3). However, large artifacts can appear in the fixed pre-frontal channels due to blinking eyes, which can be eliminated online by properly setting the rejection amplitude level of the signal.

Two additional electrodes are free and can be placed adequately according to the type of visual stimulation, such as at Oz for pattern-reversal VEPs and at Pz or lateral parieto-occipital electrodes for motion-onset VEPs [3, 5, 8]. Also, the fixed pre-frontal electrodes can be replaced with free electrodes for higher flexibility of the 4-channel VEP recordings. The reference electrode and the electrode for noise suppression of the recorded signal (Czech Technical University in Prague—patent CZ 302,454) are placed on the opposite sides of an earlobe clip.

#### VEP recording settings

**Fig. 4:**
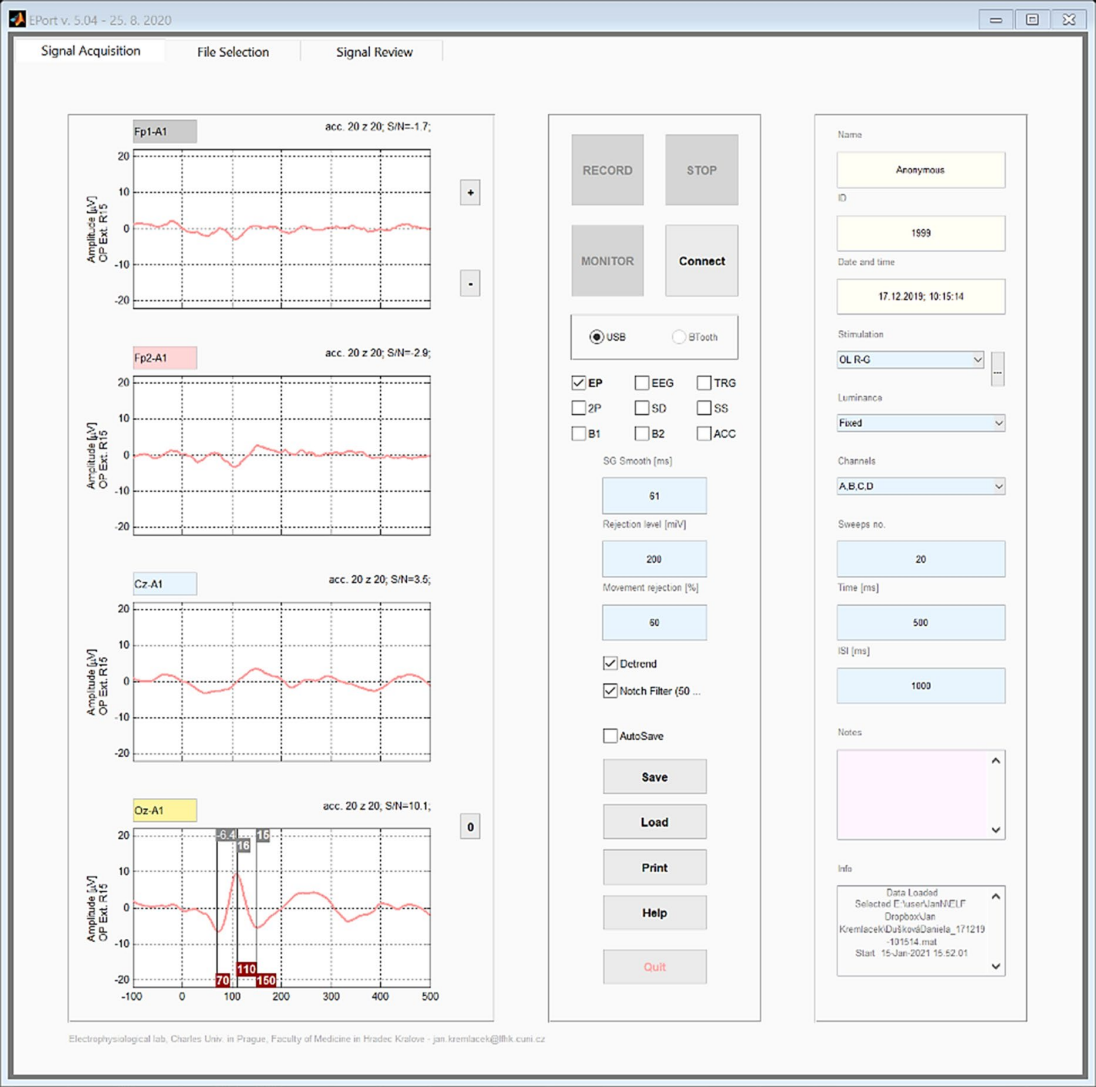
Display of average pattern-reversal VEP from the 20 single responses Oz-A1 lead has the dominant positivity at 110 ms and inter-peak amplitudes of 16 and 15 µV. In the right part of the screen, VEP recording settings are available

Before each VEP examination, the used channels and visual stimulation are selected from a menu along with the number of single VEP sweeps (EEG segments for averaging), their duration, ISI, levels for artifact rejections, and signal smoothing with a Savitzky–Golay filter. Additionally, “notch filter” and “detrend” functions can also be used. The number of accepted non-rejected single VEPs and the average VEP is displayed for each channel. It is also possible to visualize continuous EEG and some statistical characteristics of the signal, such as the signal-to-noise ratio, standard deviation, and the significant peaks/segments of the average VEPs. The layout of the whole display is shown in Fig. 4. All single VEPs are saved, and any manipulation of data is available off-line.

The noise content in the recorded EEG signal is quite low thanks to the special noise-reduction method, so a sufficient signal-to-noise ratio in the averaged VEPs is achieved with a low number of single VEPs. In most of our experiments with this VEP device, only 20 single VEP sweeps were averaged. This significantly decreases the duration of one average VEP acquisition (usually 20 s only), which can help to improve the quality and reliability of the average VEPs (fewer blinks, motion artifacts, lower tiredness—better concentration).

#### Evaluation of VEPs

**Fig. 5:**
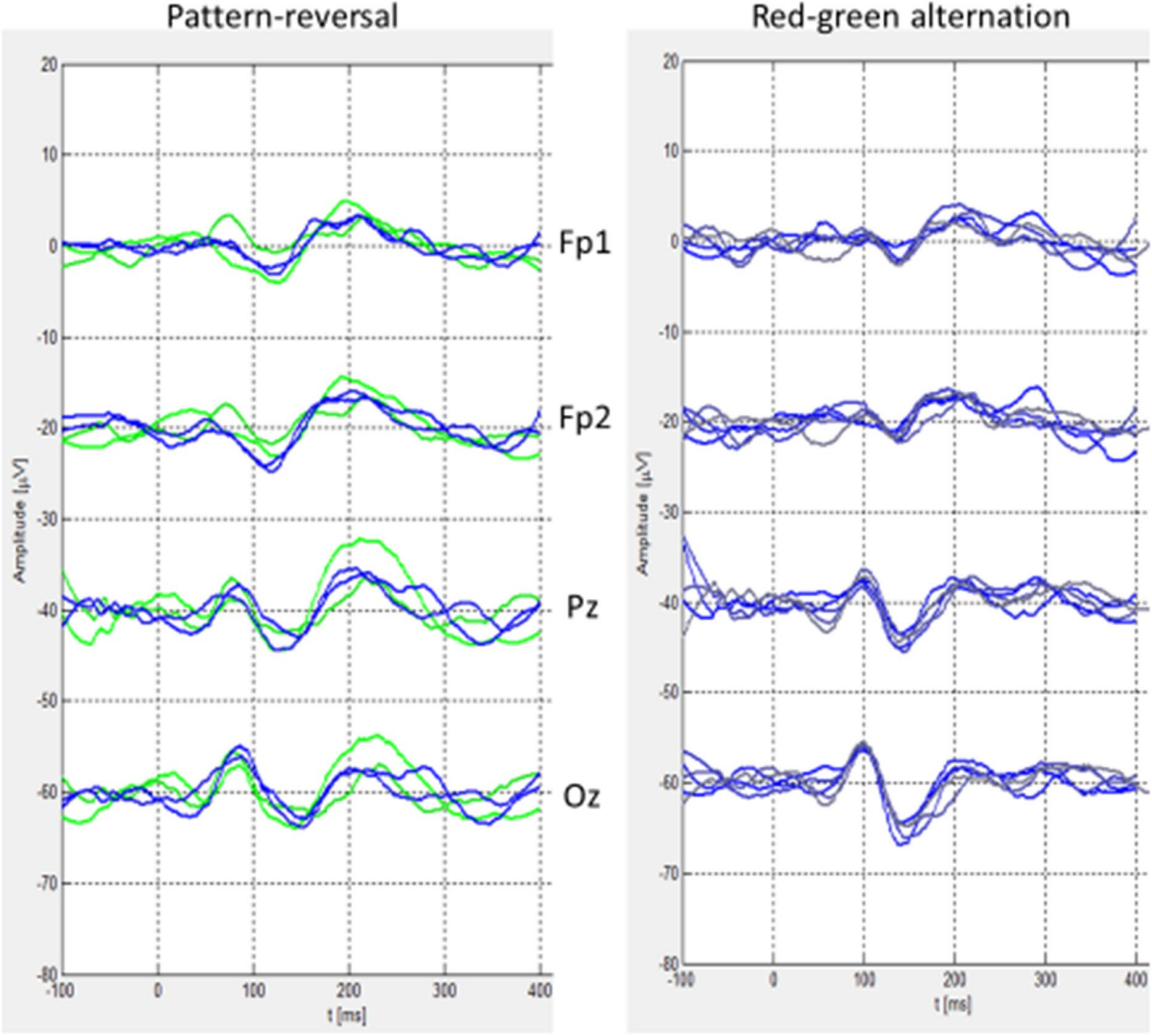
Example of overlapping VEPs for intra-/inter-individual comparisons Twice-repeated monocular VEPs are displayed (left eye in green/gray, right eye in blue). The shape of pattern-reversal and red-green alternation VEPs is similar, with slightly longer latencies in red-green VEP. Pattern-reversal has here a detectable response (negativity) in pre-frontal electrodes (upper two)

During recording, visualization of the significance of emerging peaks can be used to manually terminate the recording. In off-line evaluation, it is possible to mark significant peaks. Values of their latencies and amplitudes are saved, and a summarized table appears in the printed protocol of the VEP record. It is also possible to display and print out up to nine overlapped VEP records for visual inspection of either interocular differences, intra-individual stability of the repeated VEP recordings, or inter-individual differences (Fig. 5). In the case of visually evoked cognitive potentials, target and non-target responses are displayed online in different colors. Saved VEPs can be re-evaluated repeatedly with different settings for smoothing and rejection criteria.

### “Standard” VEP examination at our laboratory (SGlab system) with which the VEPpeak was compared

Our laboratory (founded in 1969) uses some long unchanged stimulation parameters that partially differ from those recommended by ISCEV. The differences between the used stimulations concerned stimulus luminance and spatial frequencies of checkerboard pattern-reversal (see below).

*VEP recordings* are performed in a Faraday cage, and they include unipolar derivations (with the right ear lobe reference) from the midline Oz, Pz, Cz and Fz, and Ol and Or (5 cm to the left and right from the Oz position). The lateral recording sites are used because the N2 peak of the motion-onset VEPs can be lateralized (due to different specialization of hemispheres to motion-perception) toward the temporo-occipital cortex [8].

*Visual stimuli* are generated using VSG 2.5 (CRS Ltd., UK) on a 21″ Iyama CRT monitor (Japan) with a vertical frequency of 105 Hz. The stimulus field subtends 37 × 28 deg with a viewing distance of 0.6 m, and the average luminance for all stimuli is 17 cd/m^2^. Using this rather low luminance is based on our own experience and some recommendations, e.g., [11], leading to increased examination sensitivity by optic nerve pathology. The correct visual fixation is monitored via an infrared CCD camera. In the *pattern-reversal stimulation*, black-white checkerboards with element sizes of 40’, 20’, and 10’ (standard set for about normal visual acuity) and with 96% contrast according to Michelson are reversing at a frequency of 2 reversals/s.

For the motion-onset VEPs, *radial motion* (“expansion/contraction”) of low contrast (10%) concentric circles with sinusoidal luminance modulation. The spatial frequency structure decreases, and the motion velocity increases from the center (fixation point) toward the periphery respecting the size of the retinal receptive fields and the sensitivity to motion velocity across the retina [12]. The moving stimuli have timing with 200 ms of motion, followed by 1 s ISI. For details of the standard methods in our laboratory, see, e.g., [13].

### Examined healthy subjects and patients

Several pilot studies in healthy subjects and patients (groups 1–5) were already performed with the VEPpeak to verify:

- *The reliability of the new device (groups 1, 2)*
- *Comparability of the recorded VEPs with our standard laboratory equipment (group 3)*
- *Feasibility and sensitivity of the VEP examination with the VEPpeak in neuro-ophthalmological patients (groups 4, 5)*

*Group 1*: The first study with **20 healthy volunteers** (21–24 years old) was oriented to compare parameters of VEPs acquired with the built-in LED visual stimulator.

*Group 2*: In a larger **group of 51 non-experienced healthy subjects** (20–26 years old), statistical characteristics of the VEPs were tested, using the portable device with the external stimulator. They were tested outside the laboratory in different variable environments to prove the device’s usability in almost any condition.

*Group 3*: After the introduction of the external stimulation, a group of **15 healthy subjects** (eight women and seven men aged 22–25 years) was tested to compare differences between parameters of VEPs recorded with the use of the mobile device (VEPpeak) and VEPs recorded with our standard laboratory equipment.

*Group 4*: **Fifty-two patients** were examined from the departments of ophthalmology (e.g., with suspected optic neuritis, compression or trauma of the optic nerve, glaucoma, amblyopia), from neurology (with suspected Multiple Sclerosis), and the department of infectious diseases (with neuroborreliosis influencing optic nerves). After standard VEP examination in our laboratory, also the portable device was used to record monocular pattern-reversal VEPs (60’ and 15’ check size) and motion-onset VEPs and the results of both examinations were compared.

*Group 5*: Examination of VEPs using the portable device was done in **24 immobile patients with Multiple Sclerosis** (62 ± 10 years).

Thus, altogether the functions of the portable VEP device have been tested in 86 healthy experimental subjects and 76 neuro-ophthalmological patients.

### Statistical analysis

The data were statistically processed with R software version 3.6.2 using the “nortest,” “ggplot2” and “BlandAltmanLeh” packages (R: A language and environment for statistical computing. R Foundation for Statistical Computing, Vienna, Austria).

The Anderson–Darling test for normal distribution was performed. Depending on the result, either a paired Student’s *t* test or the Wilcoxon signed-rank test was used to evaluate the differences between the VEP parameters from the VEPpeak and standard laboratory examination (SGlab system). For comparison of the diagnostic conformity of the VEPs from the portable device and standard examination, evaluation of the Pearson correlation coefficient “r” (coefficient of determination “r^2^”) was used but also Bland–Altman plots [14] and “concordance correlation coefficients” [15], which are recommended to illustrate the agreement between a new method with an existing method (alignment between instruments) [16]. Besides the basic statistical characteristics of the compared data in the text, more details (taking into consideration their rather asymmetric distributions) are included in Supplementary Material.

## Results

### VEPs recorded with built-in LED visual stimulation

The tests were done with 20 healthy volunteers—**group 1** specified in Methods. They provided satisfactorily large VEP amplitudes, with relatively low variability (see variation coefficients) and low variability of latencies compared to flash VEPs as it is shown in Table 1. The results showed that pattern-reversal, motion-onset, and also red/green LED visual stimuli might be well used for diagnostics with VEPpeak.

**Table 1:** VEP parameters with built-in LED visual stimulation

Stimulation	Lead	Latency [ms]	Var. coeff. [%]	Amplitude [µV]	Var. coeff. [%]
Reversal 4°	Oz	95 ± 11	12	4.3 ± 1.3	30
R/G alternat	Oz	99 ± 10	10	4.7 ± 1.6	34
Flash 1 Hz	Oz	103 ± 22	21	7.1 ± 3.6	51
Motion-onset	Pz	158 ± 15	9	4.9 ± 1.9	39

Dominant peak latency is specified in each VEP type (P100 in pattern-reversal, positivity in red/green alternation, positivity in flash VEP and N2 peak in the motion-onset VEP). Amplitudes represent mean inter-peak amplitudes (average of two amplitudes of the dominant peak)

Flash VEPs had the largest latency variability with a variation coefficient of over 20%. However, they remain part of the standard set of VEPs for the mobile device since high luminance flashes sometimes represent the only effective stimuli for acquiring detectable cortical responses in severely affected vision or babies. Motion-onset VEPs with low latency variability represent the obligatory part of our VEP examination because they test the magnocellular system and the dorsal stream of the visual pathway quite selectively [8].

### VEPs recorded with external visual stimulation

Introducing an external stimulator led to the magnification of the pattern-reversal and motion-onset VEPs and decreased their variability. In the **group 2** of 51 healthy subjects, variation coefficients of the dominant peak latencies of pattern-reversal VEPs from the portable device decreased below 10%, which is quite comparable with the VEPs recorded in much more constant laboratory conditions. Latencies and amplitudes distribution of all recorded VEPs is provided in Supplementary Material 1.

### Comparison of VEPpeak with our standard laboratory equipment

We tested the parameters of the acquired VEPs (**group 3**) with the mobile device in a normal environment and compared them with the parameters of VEPs recorded in our standard laboratory conditions in a Faraday cage. Comparable parameters of visual stimulations were used in both examinations performed during one joint session without any change of the recording electrodes. Two repeated VEPs examinations in **group 3** were done in a 1-month interval, and monocular VEPs from the dominant eye with normal visual acuity (with correction if needed) were recorded twice in each of the two examinations.

There were no significant differences in VEP parameters among the two repeated sessions showing their good reproducibility. The average results from the second session are summarized in Table 2. Full statistical analysis is provided in Supplementary Material 2.

**Table 2:** Comparison of VEP parameters in healthy subjects – portable device vs. standard lab

VEP type	Lead	Portable device				Standard laboratory			
		Lat [ms]	Var. coeff. [%]	Amp [µV]	Var. coeff. [%]	Lat [ms]	Var. coeff. [%]	Amp [µV]	Var. coeff. [%]
**Pattern rev. 60´**	Oz	106 ± 8	8	13 ± 5	38	108 ± 5	5	15 ± 6	40
**Pattern rev. 15´**	Oz	111 ± 8	7	12 ± 6	50	113 ± 5	4	16 ± 9	56
**Motion-onset**	Pz	153 ± 12	8	8 ± 3	37	149 ± 10	7	10 ± 3	30
**Cognitive EP**	Cz	335 ± 24	7	23 ± 9	39	368 ± 24^a^	7	18 ± 7^1^	39

^a^Not comparable cognitive stimuli with the portable device

Dominant peak parameters are specified as in Table 1. P300 (P3b) parameters were evaluated in cognitive evoked potentials

Except for visually evoked cognitive potentials (in which the cognitive stimulation in the laboratory was not comparable with the LED cognitive stimulus in the VEPpeak), all other kinds of VEPs had larger amplitudes and lower variability of latencies in standard laboratory conditions with a larger stimulus field and precisely constant physical parameters of the environment, including the electromagnetic shielding in the Faraday cage. Despite some latency differences dependent on the stimulus parameters and recording conditions, VEPs from the mobile device are robust enough to be suitable for diagnostic purposes.

### Results of the pilot testing of the diagnostic sensitivity of the VEPpeak

In **group 4**, we have examined 52 patients (104 eyes) to verify whether the VEP results of the portable device agree with the diagnostic conclusions based on the standard VEP examination. Data and statistical parameters of all evaluated electrophysiological data for each examined eye are provided in **Supplementary Material 3**. Summarized average values in **Table ****3** show that there are small but significant differences in paired comparison. Systematically smaller amplitudes and shorter latencies of VEPs from the portable device result highly probably from the smaller stimulus field and higher luminance of stimuli (40 cd/m^2^ vs. 17 cd/m^2^ in laboratory conditions). However, all parameters displayed close correlation (Pearson’s r = 0.57–0.81) and Bland–Altman plots [14] and “concordance correlation coefficients” [15] (in **Supplementary Material 3**) signalize good agreement between both VEP examination methods.

**Table 3:** Comparison of VEP parameters from standard examination (SGlab) and portable device (VEPpeak) in 52 neuro-ophthalmological patients

Stimulation	Latency [ms]	Var. coeff. [%]	Amplitude [µV]	Var. coeff. [%]
SGlab R40´	111 ± 11	10	10 ± 6	60
VEPpeak R60´	103 ± 12	12	7 ± 5	71
SGlab R20´	117 ± 13	11	10 ± 6	60
VEPpeak R15´	112 ± 14	12	7 ± 5	71
SGlab MO	163 ± 18	11	8 ± 4	50
VEPpeak MO	168 ± 18	11	7 ± 4	57

R40´—pattern-reversal 40´ and accordingly R60´, R20´, R15´; MO – motion-onset VEP

**Fig. 6:**
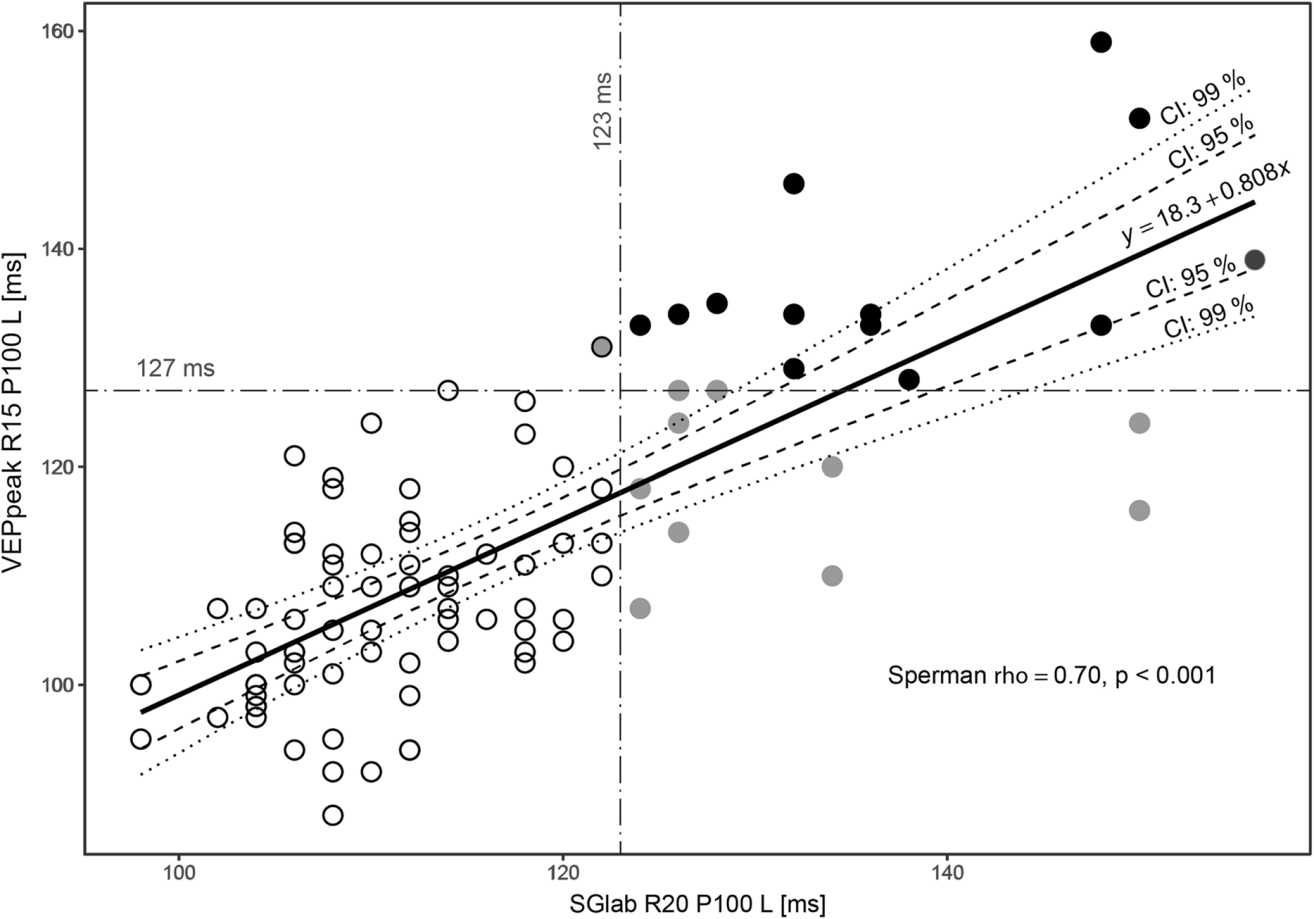
Correlation between pattern-reversal VEP latencies from standard laboratory examination and VEPpeak in 94 eyes of neuro-ophthalmological patients

Figure 6 shows the distribution of the most sensitive diagnostic criterion—the P100 latency of the pattern-reversal 15’ (VEPpeak) or 20’ (standard laboratory system SGlab). Only 8 out of 94 eyes (= 8.5%) in which reliable VEPs were earned (in low visual acuity they were not detectable) are in the right bottom quadrant of the graph—gray points are over the limit (> M + 2SD = 123 ms) for SGlab but below the limit for VEPpeak, another two have border latency values. This signalizes a slightly lower sensitivity (detection of a pathology) of the VEPpeak, which might depend on the little bit higher variability of VEP latencies from the portable device (Table 2) or it is possibly because of the specified suspected higher sensitivity of VEPs in the lower stimulation luminance [11]. There was also one case/eye (left upper quadrant) in which the pathology of the optic pathway was detected only with the portable device.

Similar results were also in comparison of the other evaluated VEP parameters. Altogether, in about 90% of the examined eyes, the diagnostic conclusions from both examinations were identical.

In the group of 24 immobile patients with multiple sclerosis (**group 5**), it was confirmed that the VEP examination with the portable device is feasible in patients who could hardly undergo the standard VEP recordings in a normal electrophysiological laboratory. In all these severely affected patients, significantly prolonged pattern-reversal or motion-onset VEP latencies were found in at least one eye. The mean latency value for pattern-reversal 60’ P100 peak was 142 ± 16 ms (upper limit 122 ms), and that for motion-onset N2 peak 201 ± 21 ms (upper limit 177 ms). More details are in **Supplementary Material 4**.

We also tested the device by examining three comatose babies with severe perinatal post-traumatic/hypoxic brain involvement in the ICU. Using flash stimuli over closed eyelids, we successfully verified that there was the functioning projection by the visual pathway up to the primary visual cortex, resulting in detectable VEPs but with prolonged latencies. Such urgent diagnostic examination in the ICU would probably not be simply possible with standard VEP equipment.

## Discussion

The development of the portable VEP device took about 10 years but here we provide only a description of the properties and parameters of the 5th latest version of the prototype which is now ready for clinical use in neuro-ophthalmology. The most difficult technical problem was the elimination of stimulation artifacts from the recorded signal caused by the current for the LED stimulator.

The testing of a large spectrum of possible visual stimuli should continue since, e.g., the proposed long-term VEP monitoring for possible detection of CNS fatigue and dysfunctions requires the use of adequate stimulation that also activates pre-frontal areas. Then, only the dry electrodes on the forehead might be sufficient without any other electrode montage. This would simplify using the device for repeated self-examinations of VEPs in some CNS dysfunctions that change over time—e.g., in some chronic encephalopathies (uremic, hepatic), in changes of glycemia or in monitoring of effects of psychofarmacs.

VEPs to red/green alternation are not diagnostically used yet because of their more difficult interpretation. They display larger inter-individual shape variability and distinct resistance to the pathology of the visual pathway. But they are quite robust and recordable also in pre-frontal areas.

The achieved low inter-individual variability of VEPs from VEPpeak allows the creation of relatively narrow latency norms, which is an important prerequisite for good diagnostic usability of the device, even in improvised conditions outside the standard laboratories.

In about 90% of the examined eyes, the diagnostic conclusions from VEP examinations with the new portable device and the standard VEP equipment were identical, which can be considered a good recommendation for using the portable device for a basic screening (monitoring) of a visual pathway pathology. It should still improve when more precise norms from a larger group of control subjects (age-dependent norms) as we have it for the SGlab system [13] will be created for the VEPpeak.

Our first results with cognitive potentials in schizophrenics (in preparation) show that the mobile device can also be used to examine patients who are not fully cooperative. Their potential anxiety decreases when they are examined in an environment they are familiar with (instead of a laboratory setting).

To our knowledge, there are recently numerous portable EEG devices in various headsets, but they are equipped with neither suitable visual stimulators nor software for clinical diagnostic VEP examination. Few exceptions exist, e.g., RETeval by “LKC,” a portable handheld ERG/VEP device providing only flash VEPs examination (https://lkc.com/products/reteval-2/). Just now, a new solution using smart glasses or Google Cardboard for pattern-reversal stimulation (instead of a PC monitor) combined with the small OpenBCI Cyton Board appeared [17]. Thus, it looks as though the need to provide a mobile VEP examination is already being understood.

Improvised VEP examinations outside a standard laboratory using existing stationary VEP equipment are not a common practice, mainly because of troubles with montage/demontage and transport. Therefore, a simple fully portable device can be helpful in many situations. Based on our first experience, we believe that the device might also extend the detection of such CNS and visual pathway dysfunctions that might not be sometimes recognizable by CT or MRI, such as in neuroborreliosis [18] various encephalopathies [19, 20] or HIV positive patients [21]. However, a larger multicentric studies are needed for verification of its practical usability.

## Conclusions

The VEPpeak device offers full portability and a 4-channel high-quality signal that is noise-resistant and free of motion artifacts. It has large visual stimulation facilities, ISCEV VEP standard compatibility, the possibility of long-term VEP (self-) monitoring, quick and simple use, and a low price. These properties might help extend the diagnostic use and improve the accessibility of VEP examination, of which the importance currently seems to be underestimated.

## Supplementary Information

Below is the link to the electronic supplementary material.

Supplementary file1 (DOCX 1140 KB)

## Data Availability

The dataset is obtainable from the corresponding author upon reasonable request. More information is available at “VEPpeak.com.”

## Code availability

The analysis script is available from the corresponding author upon reasonable request.

## Declarations

### Statement on the welfare of animals

This report does not contain any studies with animals performed by any of the authors.
