# Supplementary material for: VEP examination with new portable device
Source: Doc Ophthalmol. 2022 Nov 27;146(1):79–91. doi: 10.1007/s10633-022-09911-w (PMC9911502; doi:10.1007/s10633-022-09911-w)

**Supplementary material 1 (results of the pilot study in the group 2)**

**VEP LATENCY distribution in 51 subjects for pattern reversal R60´and R15´, motion-onset and cognitive EP**


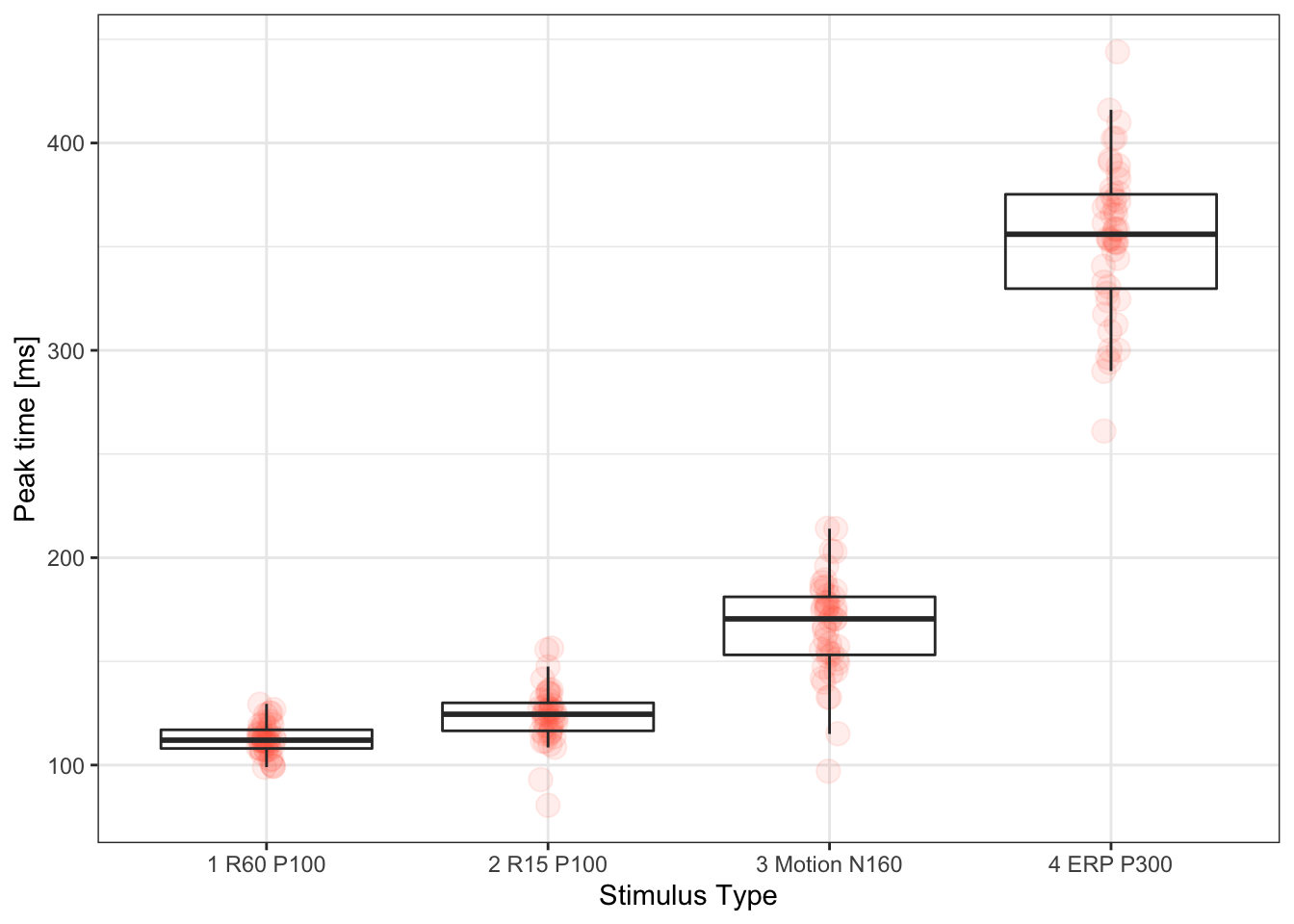


**VEP AMPLITUDE distribution in 51 subjecs for pattern reversal 60´and 15´, motion-onset and cognitive EP**


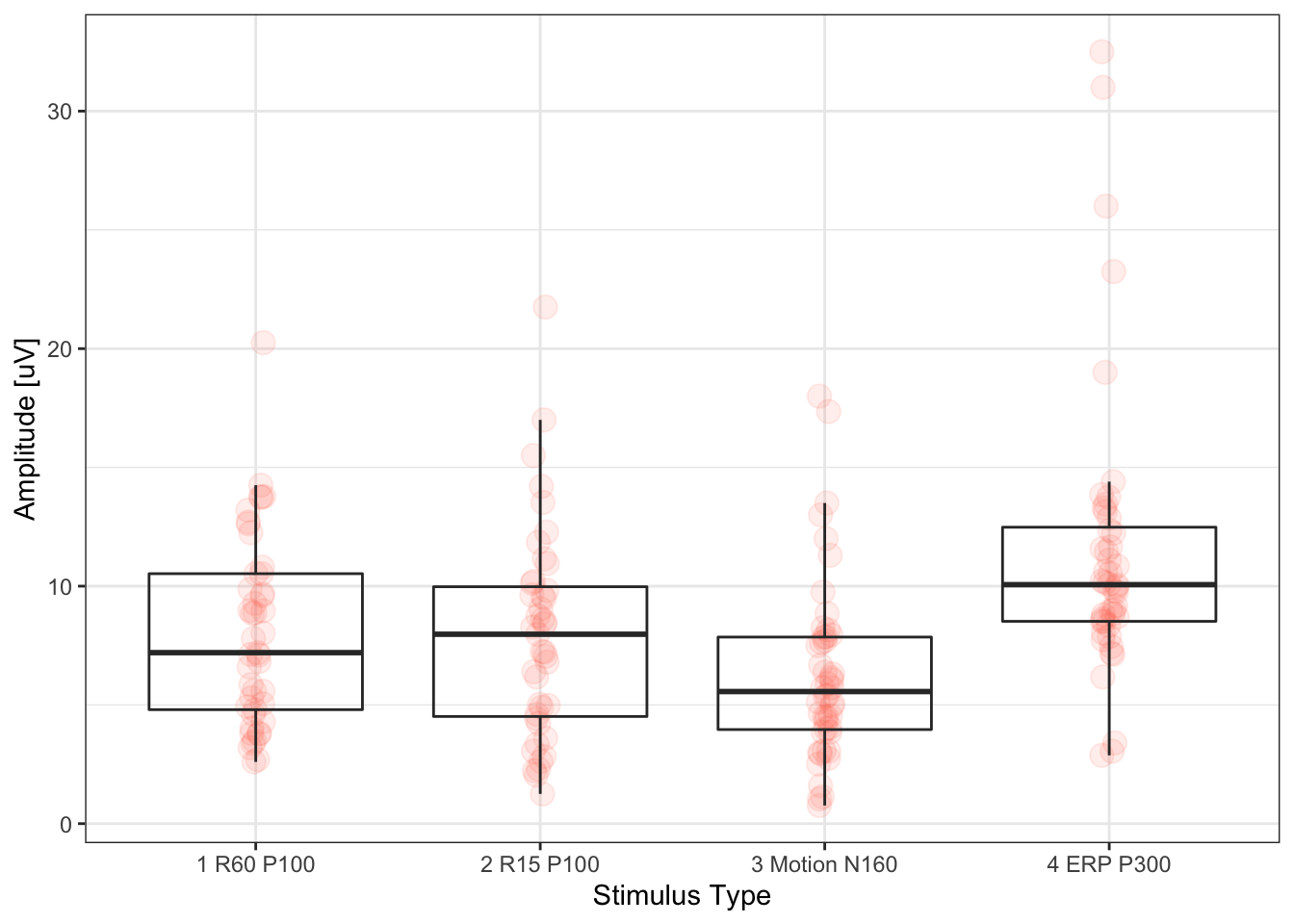


**Supplementary material 2 (results of the pilot study in the group 3)**

**Comparison of VEP parameters (portable device) in 15 subjects between the 1st and 2nd session**

(interval between the two sessions – one month)

**No statistically significant differences were found between the data of the two sessions.**

|  |  | 25q | Median | 75q | Mean | SD |
| --- | --- | --- | --- | --- | --- | --- |
| 1. session R60 P100 L [ms] |  | 100.0 | 101.5 | 110.5 | 103.96 | 6.73 |
| 2. session R60 P100 L [ms] |  | 101.5 | 102.0 | 104.5 | 105.04 | 7.85 |

|  |  | 25q | Median | 75q | Mean | SD |
| --- | --- | --- | --- | --- | --- | --- |
| 1. session R60 P100 A [uV] |  | 9.18 | 11.88 | 17.50 | 12.97 | 5.43 |
| 2. session R60 P100 A [uV] |  | 8.43 | 12.05 | 17.75 | 12.93 | 4.66 |

|  |  | 25q | Median | 75q | Mean | SD |
| --- | --- | --- | --- | --- | --- | --- |
| 1. session R15 P100 L [ms] |  | 103.5 | 104.5 | 115 | 108.35 | 7.93 |
| 2. session R15 P100 L [ms] |  | 107.0 | 109.0 | 112 | 111.92 | 7.51 |

|  |  | 25q | Median | 75q | Mean | SD |
| --- | --- | --- | --- | --- | --- | --- |
| 1. session R15 P100 A [uV] |  | 6.12 | 10.88 | 14.75 | 11.04 | 5.94 |
| 2. session R15 P100 A [uV] |  | 6.45 | 11.95 | 18.75 | 12.50 | 6.62 |

|  |  | 25q | Median | 75q | Mean | SD |
| --- | --- | --- | --- | --- | --- | --- |
| 1. session motion N160 L [ms] |  | 146.50 | 149.50 | 156.88 | 151.21 | 8.01 |
| 2. session motion N160 L [ms] |  | 146.00 | 151.75 | 154.38 | 153.25 | 11.70 |

|  |  | 25q | Median | 75q | Mean | SD |
| --- | --- | --- | --- | --- | --- | --- |
| 1. session motion N160 A [uV] |  | 5.42 | 6.26 | 9.02 | 7.34 | 2.51 |
| 2. session motion N160 A [uV] |  | 5.86 | 7.76 | 9.41 | 7.73 | 2.34 |

|  |  | 25q | Median | 75q | Mean | SD |
| --- | --- | --- | --- | --- | --- | --- |
| 1. session P300 L [ms] |  | 313.38 | 322.5 | 342.50 | 330.75 | 24.52 |
| 2. session P300 L [ms] |  | 318.62 | 329.5 | 350.12 | 335.18 | 23.53 |

|  |  | 25q | Median | 75q | Mean | SD |
| --- | --- | --- | --- | --- | --- | --- |
| EPort 1.v. P300 A [uV] |  | 17.25 | 19.50 | 23.19 | 21.19 | 6.16 |
| EPort 2.v. P300 A [uV] |  | 18.81 | 23.12 | 24.94 | 23.05 | 8.80 |

**Supplementary material 3 (results of the pilot study in the group 4)**

SGLab vs. VEPpeak

Comparison of results in 52 neuro-ophthalmological patients

## SGlab R40´ vs. VEPpeak R60´ peak time

*Two tail tests* Anderson-Darling test: normality = FALSE

**SIGNIFICANT** paired difference between groups, Wilcoxon p= 1.569693e-12

Effect size and confidence limits, d = 1.09 [ 0.78 1.39 ]

Power of study with aforementioned effect, pwr = 1

### Description

|  | N | 25q | Median | 75q | Mean | SD | Min | Max | NaN |
| --- | --- | --- | --- | --- | --- | --- | --- | --- | --- |
| SGlab R40´ P100 L [ms] | 97 | 104 | 108 | 116 | 111.48 | 11.21 | 96 | 158 | 0 |
| VEPpeak R60´ P100 L [ms] | 97 | 94 | 101 | 108 | 102.75 | 11.84 | 72 | 137 | 0 |
| Diff. | 97 | 3 | 9 | 14 | 8.73 | 9.24 | -20 | 29 | 0 |

### Correlation graph


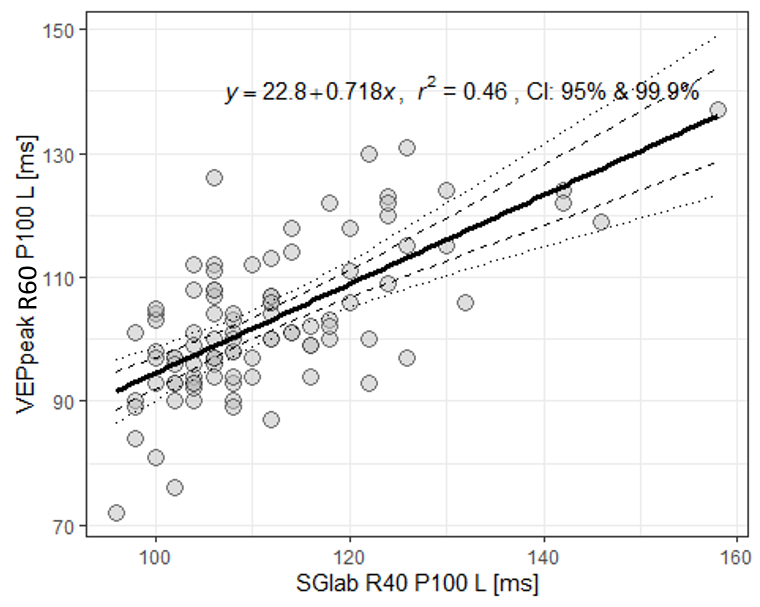


### Bland-Altman plot

Concordance Correlation Coefficient: 0.5318709 [ 0.3954891 0.6453047 ]

Critical difference is 18.1201


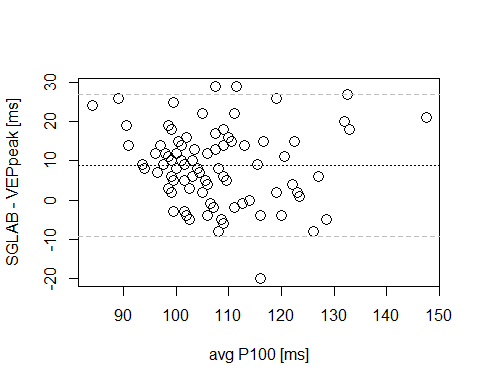


## SGlab R20´vs. VEPpeak R15´ peak time

### Paired test

*Two tail tests* Anderson-Darling test: normality = FALSE

**SIGNIFICANT** paired difference between groups, Wilcoxon p= 0.0001701087

Effect size and confidence limits, d = 0.56 [ 0.27 0.85 ]

Power of study with aforementioned effect, pwr = 0.97

### Description

|  | N | 25q | Median | 75q | Mean | SD | Min | Max | NaN |
| --- | --- | --- | --- | --- | --- | --- | --- | --- | --- |
| SGlab R20´ P100 L [ms] | 94 | 108 | 112 | 122 | 116.55 | 12.89 | 98 | 156 | 0 |
| VEPpeak R15´ P100 L [ms] | 94 | 103 | 110 | 120 | 112.45 | 13.91 | 88 | 159 | 0 |
| Diff. | 94 | -2 | 4 | 10 | 4.11 | 9.55 | -15 | 34 | 0 |

### Correlation graph


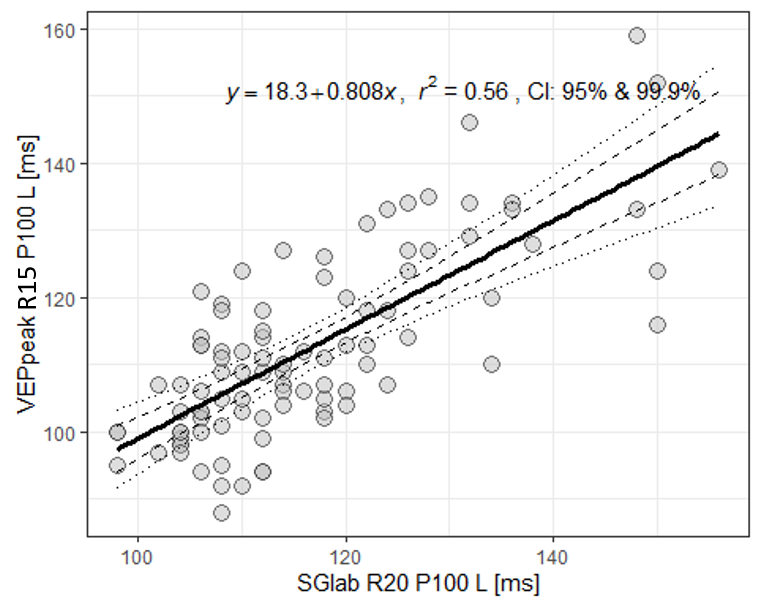


### Bland-Altman plot

Concordance Correlation Coefficient: 0.7151121 [ 0.5958558 0.8035106 ]

Critical difference is 18.71701


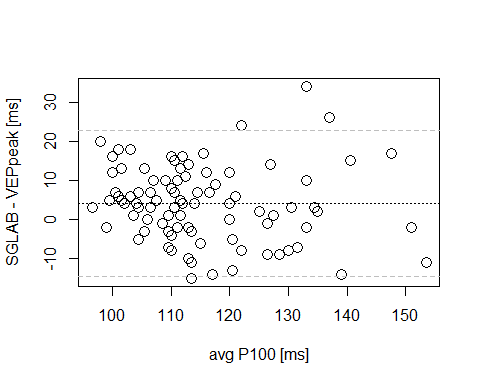


## SGlab MO vs. VEPpeak MO peak time

### Paired test

*Two tail tests* Anderson-Darling test: normality = TRUE

**SIGNIFICANT** paired difference between groups, ttest p= 0.002349777

Effect size and confidence limits, d = 0.44 [ 0.16 0.72 ]

Power of study with aforementioned effect, pwr = 0.87

### Description

|  | N | 25q | Median | 75q | Mean | SD | Min | Max | NaN |
| --- | --- | --- | --- | --- | --- | --- | --- | --- | --- |
| SGlab N160 L [ms] | 98 | 152.00 | 164.0 | 172.00 | 162.59 | 17.65 | 112 | 208 | 0 |
| VEPpeak N160 L [ms] | 98 | 154.25 | 168.5 | 180.00 | 167.88 | 18.21 | 129 | 206 | 0 |
| Diff. | 98 | -15.00 | -5.0 | 4.75 | -5.29 | 16.75 | -72 | 35 | 0 |

### Correlation graph


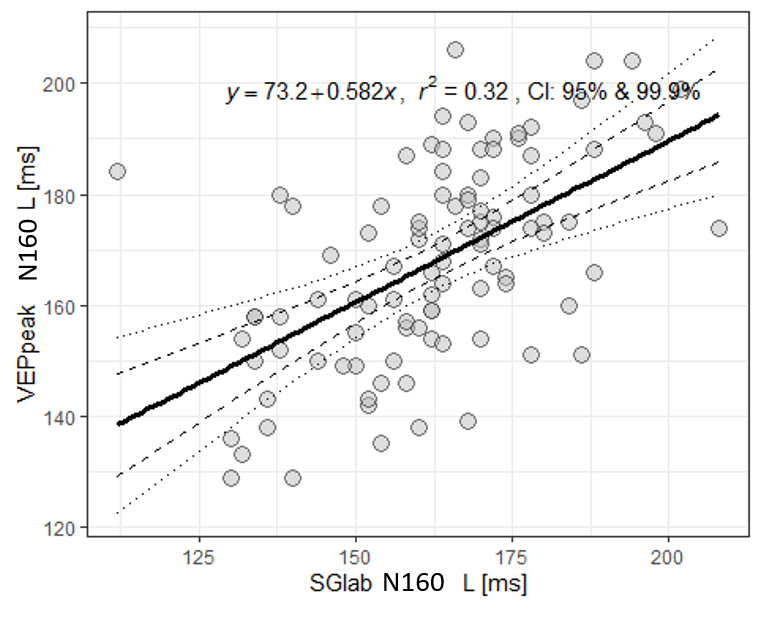


### Bland-Altman plot

Concordance Correlation Coefficient: 0.5377402 [ 0.3410629 0.6893139 ]

Critical difference is 32.82483


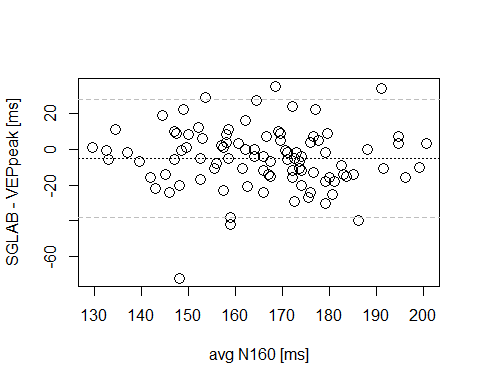


## SGlab R40´ vs. VEPpeak R60´ amplitude

### Paired test

*Two tail tests* Anderson-Darling test: normality = FALSE

**SIGNIFICANT** paired difference between groups, Wilcoxon p= 1.885157e-10

Effect size and confidence limits, d = 0.93 [ 0.64 1.22 ]

Power of study with aforementioned effect, pwr = 1

### Description

|  | N | 25q | Median | 75q | Mean | SD | Min | Max | NaN |
| --- | --- | --- | --- | --- | --- | --- | --- | --- | --- |
| SGlab R40´ P100 A [uV] | 104 | 6 | 9.0 | 13 | 9.84 | 5.65 | 0 | 26 | 0 |
| VEPpeak R60´ P100 A [uV] | 104 | 4 | 6.5 | 10 | 7.39 | 5.20 | 0 | 25 | 0 |
| Diff. | 104 | 1 | 2.0 | 4 | 2.44 | 3.49 | -10 | 12 | 0 |

### Correlation graph


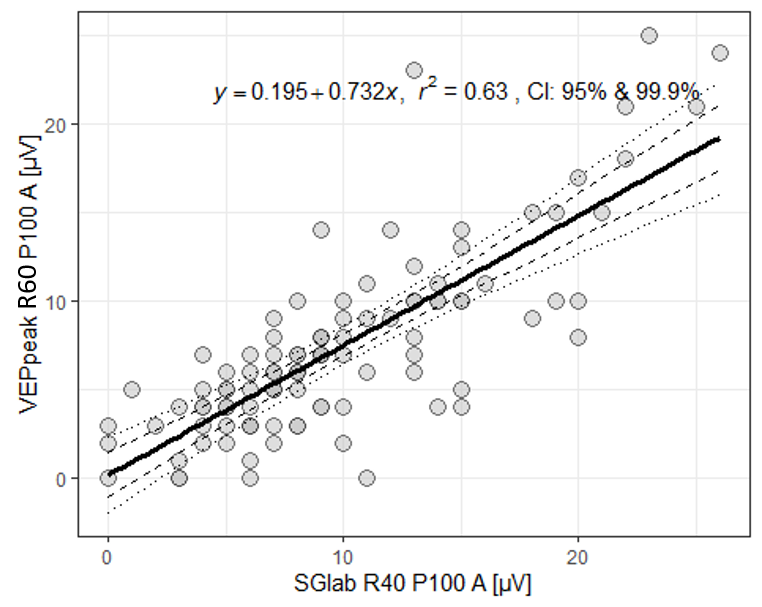


### Bland-Altman plot

Concordance Correlation Coefficient: 0.7211457 [ 0.5916341 0.8143736 ]

Critical difference is 6.848856


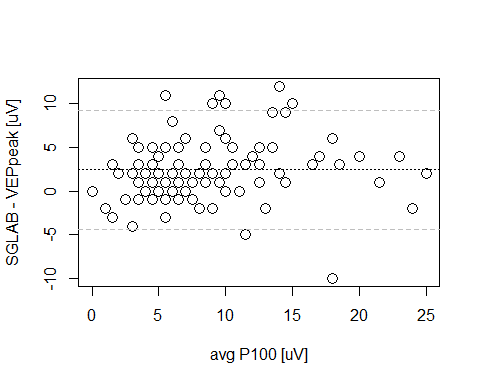


## SGlab R20´ vs. VEPpeak R15´ amplitude

### Paired test

*Two tail tests* Anderson-Darling test: normality = FALSE

**SIGNIFICANT** paired difference between groups, Wilcoxon p= 1.758405e-09

Effect size and confidence limits, d = 0.87 [ 0.59 1.16 ]

Power of study with aforementioned effect, pwr = 1

### Description

|  | N | 25q | Median | 75q | Mean | SD | Min | Max | NaN |
| --- | --- | --- | --- | --- | --- | --- | --- | --- | --- |
| SGlab R20´ P100 A [uV] | 104 | 5 | 9 | 13.25 | 9.80 | 6.42 | 0 | 28 | 0 |
| VEPpeak R15´ P100 A [uV] | 104 | 4 | 6 | 11.00 | 7.15 | 4.84 | 0 | 24 | 0 |
| Diff. | 104 | 0 | 2 | 5.00 | 2.64 | 3.78 | -4 | 13 | 0 |

### Correlation graph


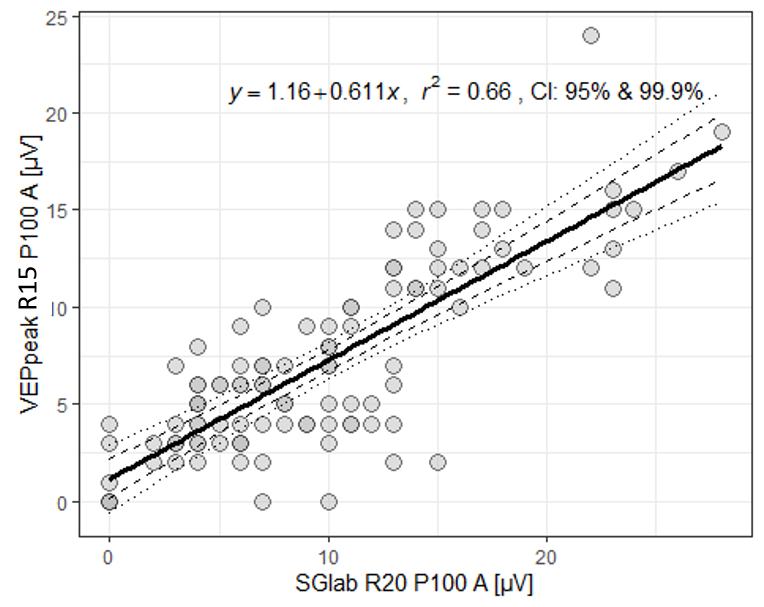


### Bland-Altman plot

Concordance Correlation Coefficient: 0.7037117 [ 0.6082267 0.7791208 ]

Critical difference is 7.409131


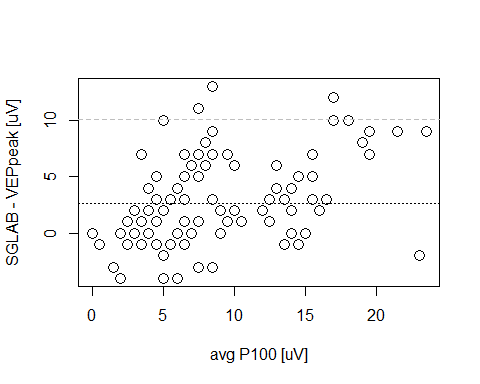


## SGlab MO vs. VEPpeak MO amplitude

### Paired test

*Two tail tests* Anderson-Darling test: normality = FALSE

**SIGNIFICANT** paired difference between groups, Wilcoxon p= 4.223358e-07

Effect size and confidence limits, d = 0.72 [ 0.44 1.01 ]

Power of study with aforementioned effect, pwr = 1

###
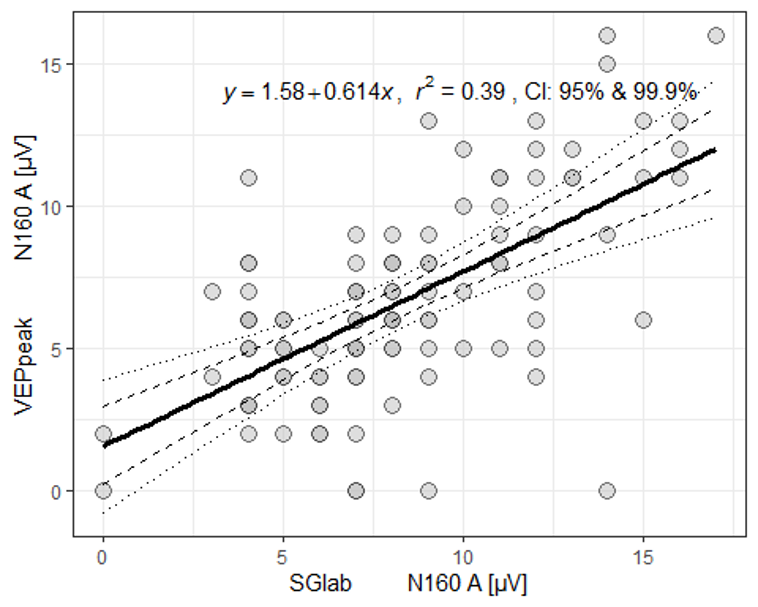
Description

|  | N | 25q | Median | 75q | Mean | SD | Min | Max | NaN |
| --- | --- | --- | --- | --- | --- | --- | --- | --- | --- |
| SGlab N160 A [uV] | 104 | 6.00 | 8.0 | 11 | 8.34 | 3.60 | 0 | 17 | 0 |
| VEPpeak N160 A [uV] | 104 | 4.75 | 6.0 | 9 | 6.69 | 3.54 | 0 | 16 | 0 |
| Diff. | 104 | 0.00 | 1.5 | 3 | 1.64 | 3.09 | -7 | 14 | 0 |

### Correlation graph

### Bland-Altman plot

Concordance Correlation Coefficient: 0.5632186 [ 0.3753617 0.7066295 ]

Critical difference is 6.057577


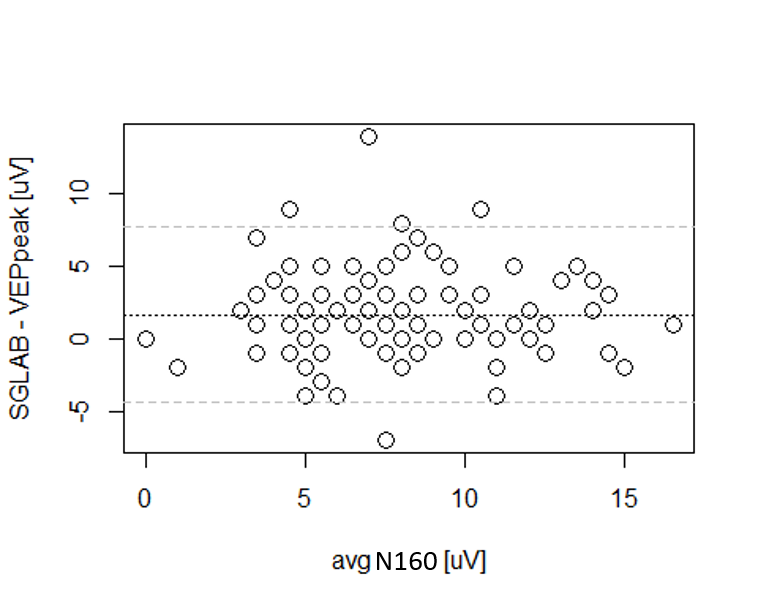


**Supplementary material 4 (results of the pilot study in the group 5)**

**Pattern-reversal R60´ and motion-onset VEP latencies in patients with Multiple sclerosis**

Right eye (RE) vs. Left eye (LE) – correlation signalizes bilateral optic nerve involvement

In both eyes the average group values of interpeak latencies are significantly prolonged, all 21 patients displayed pathological latency at least in one eye, some VEPs were not detectable (R15´ in 13 cases).


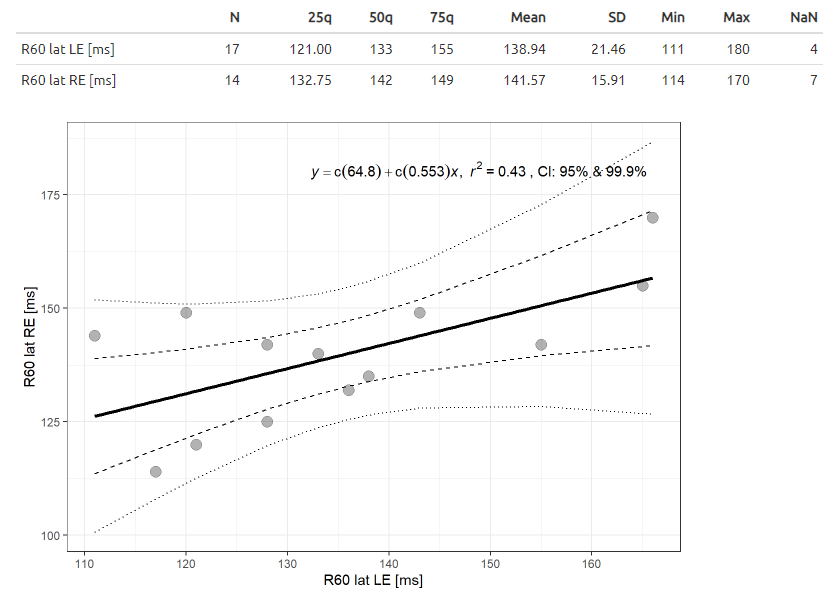


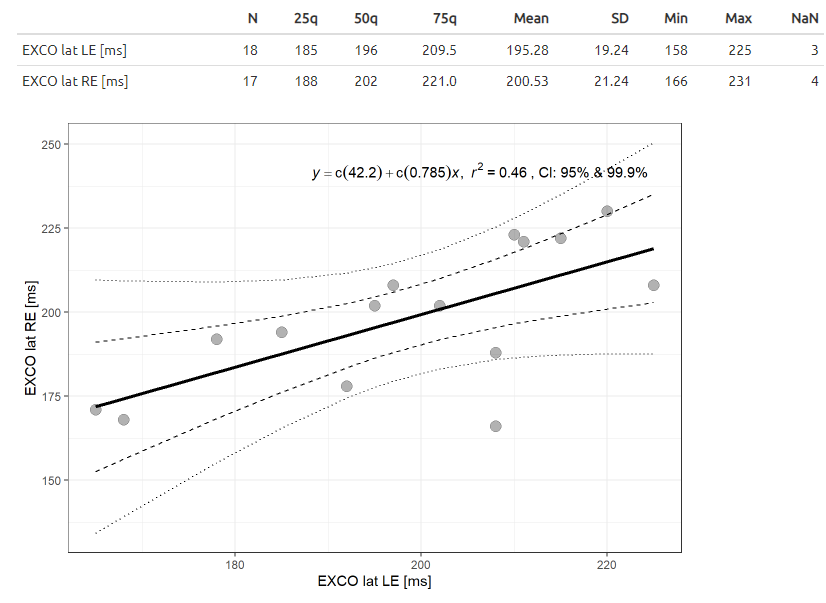

Supplement: Supplementary file 1 — Supplementary file1 (DOCX 1140 KB) [file 10633_2022_9911_MOESM1_ESM.docx]
